# Comparison of the Safety and Pharmacokinetics of ST-246® after IV Infusion or Oral Administration in Mice, Rabbits and Monkeys

**DOI:** 10.1371/journal.pone.0023237

**Published:** 2011-08-15

**Authors:** Yali Chen, Adams Amantana, Shanthakumar R. Tyavanagimatt, Daniela Zima, X. Steven Yan, Gopi Kasi, Morgan Weeks, Melialani A. Stone, William C. Weimers, Peter Samuel, Ying Tan, Kevin F. Jones, Daniel R. Lee, Shirley S. Kickner, Bradley M. Saville, Martin Lauzon, Alan McIntyre, Kady M. Honeychurch, Robert Jordan, Dennis E. Hruby, Janet M. Leeds

**Affiliations:** 1 SIGA Technologies, Corvallis, Oregon, United States of America; 2 Charles River Laboratories, Reno, Nevada, United States of America; 3 Charles River Laboratories, Quebec, Canada; Duke University, United States of America

## Abstract

**Background:**

ST-246® is an antiviral, orally bioavailable small molecule in clinical development for treatment of orthopoxvirus infections. An intravenous (IV) formulation may be required for some hospitalized patients who are unable to take oral medication. An IV formulation has been evaluated in three species previously used in evaluation of both efficacy and toxicology of the oral formulation.

**Methodology/Principal Findings:**

The pharmacokinetics of ST-246 after IV infusions in mice, rabbits and nonhuman primates (NHP) were compared to those obtained after oral administration. Ten minute IV infusions of ST-246 at doses of 3, 10, 30, and 75 mg/kg in mice produced peak plasma concentrations ranging from 16.9 to 238 µg/mL. Elimination appeared predominately first-order and exposure dose-proportional up to 30 mg/kg. Short IV infusions (5 to 15 minutes) in rabbits resulted in rapid distribution followed by slower elimination. Intravenous infusions in NHP were conducted at doses of 1 to 30 mg/kg. The length of single infusions in NHP ranged from 4 to 6 hours. The pharmacokinetics and tolerability for the two highest doses were evaluated when administered as two equivalent 4 hour infusions initiated 12 hours apart. Terminal elimination half-lives in all species for oral and IV infusions were similar. Dose-limiting central nervous system effects were identified in all three species and appeared related to high C_max_ plasma concentrations. These effects were eliminated using slower IV infusions.

**Conclusions/Significance:**

Pharmacokinetic profiles after IV infusion compared to those observed after oral administration demonstrated the necessity of longer IV infusions to (1) mimic the plasma exposure observed after oral administration and (2) avoid C_max_ associated toxicity. Shorter infusions at higher doses in NHP resulted in decreased clearance, suggesting saturated distribution or elimination. Elimination half-lives in all species were similar between oral and IV administration. The administration of ST-246 was well tolerated as a slow IV infusion.

## Introduction

Variola virus (Strain Harper DQ441430) causes human smallpox and is highly contagious with a mortality rate of approximately 30% [1]. Although smallpox was eradicated after a highly successful vaccination campaign [2], there is reason to be concerned about either deliberate or accidental re-introduction into the human population [3]. In addition, there are three other orthopoxvirus species (monkeypox virus strain Zaire NC 003310, vaccinia virus strain Western Reserve NC 006998, and cowpox virus strain Brighton Red NC 003663) that infect humans and cause significant disease [4]–[6]. While these viruses are less pathogenic than variola virus, they retain the capacity to cause serious illnesses and even death [7], [8].

There are currently no approved therapeutic treatments for orthopoxvirus infections, although cidofovir, a nephrotoxic drug that is approved for CMV retinitis, has shown activity against orthopoxviruses *in vitro* and *in vivo* in animal models [9]–[12]. Cidofovir has been administered for treatment of orthopoxvirus-related illness [13]–[16]. In order to avoid kidney toxicity and death (as described in the package insert) cidofovir must be co-administered with probenecid and hydration therapy [17]–[19]. Oral prodrugs of cidofovir are currently being developed to mitigate kidney toxicity and improve therapeutic properties of the molecule [20]. Vaccines to protect against orthopoxvirus infection have been approved by the FDA, but the high frequency of serious adverse events associated with the vaccine and relatively low risk of infection have limited their use [21]. Currently, only military personnel being deployed to areas perceived to be at high risk for bioterrorism and laboratory workers exposed to orthopoxviruses are being vaccinated [22]. If an orthopoxvirus outbreak occurred, exposed individuals would have to be treated with IV cidofovir (and likely vaccinia immune globulin (VIG)), to mitigate disease until vaccine could be deployed. Moreover, post-exposure vaccination is less effective at altering the disease course after the fourth day of infection; thus, effective antiviral treatment would be the only viable option to treat exposed individuals [23].

ST-246 (Tecovirimat: 4-trifluoromethyl-N-(3,3a,4,4a,5,5a,6,6a-octahydro-1,3-dioxo-4,6-ethenocycloprop[*f*]isoindol-2(1H)-yl)-benzamide) is a novel, orally available small molecule that specifically inhibits viral egress [24], [25]. The target of ST-246 has been identified as the product of the *F13L* gene in vaccinia virus [24], which is highly conserved among all orthopoxviruses, particularly in the region of the gene targeted by ST-246 [26]. Reported EC_50_ values against different poxviruses *in vitro* range from .007 to 0.16 µg/mL [27]. *In vivo* studies have demonstrated potent efficacy against vaccinia virus, cowpox virus and ectromelia virus [28] in mice, providing optimal efficacy at a dose of 100 mg/kg. Additional *in vivo* efficacy has been demonstrated in a ground squirrel model of monkeypox virus [29], rabbitpox virus in rabbits [30] and variola virus and monkeypox in NHP [31], [32], at daily doses of 100, 40, 10, and 3 mg/kg, respectively [33].

Oral, nonclinical safety studies have demonstrated safety through three months daily administration in mice and NHP. In NHP, the highest dose evaluated in the 3 month safety study, 300 mg/kg, was considered the No Observed Effect Level (NOEL), due to the lack of any observed effect, and was 100-fold higher than the efficacious dose in NHP of 3 mg/kg after infection with monkeypox virus [32]. Oral bioavailability has been estimated to be near 50%, with limited metabolism and largely biliary excretion observed in a mass balance study in mice [34]. In parallel with the animal efficacy studies, human safety evaluation of ST-246 has demonstrated that oral administration for 21 days is safe, with no serious adverse events having been reported after administration to healthy adults [35]. Exposure to orally administered ST-246 is dose proportional at lower doses, but absorption appears to become saturating at higher doses [34]. Steady-state appeared to be reached after 6 days of administration, consistent with the estimated 20 hour terminal elimination half-life [34]. The accumulation index was estimated to be approximately 20% for daily dosing, indicating very little accumulation at steady-state [34]. The long terminal elimination half-life and high therapeutic index readily allow for single daily oral administration in animal models of disease, as well as in humans [35].

During the short time that ST-246 has been in clinical evaluation, there have been several occasions in which ST-246 has been requested for emergency use. In two of those cases, oral administration was not the optimal route of administration. In the first case [5], the patient was a young child who had been infected with vaccinia virus after coming in close contact with his father, who had received the smallpox vaccine. The child developed severe eczema vaccinatum, and after unsuccessful treatment with Vaccinia Immune Globulin Intravenous (VIGIV), was administered ST-246. Based on the child's inability to swallow a pill and the need to use a very low dose due to his low body weight, the ST-246 was administered via a nasogastric tube [36]. In the second instance, a 20-year old male had developed progressive vaccinia after receiving cancer chemotherapy subsequent to having received the smallpox vaccine. The patient was taking ST-246 with little to no food, significantly decreasing absorption [33], [37]. In both of these cases, an IV formulation would have facilitated dose administration and simplified any required dose adjustment.

A new formulation has been developed for IV administration of ST-246. The tolerability and pharmacokinetics of this formulation have been evaluated in mice, rabbits and NHP in order to determine the optimal administration strategy. The results are compared with the pharmacokinetics observed after oral administration.

## Methods

### Materials

All materials used in the conduct of these studies were reagent grade, or higher, unless specifically noted below. The source, where a material may not be readily available, is noted.

### Study designs and animal in-life studies

#### Ethics Statement

The in-life portions of the experiments were conducted at several different laboratories, all of which conducted studies according to all Federal, State, and local guidelines for the use of animals in research and were reviewed and approved by their respective Institutional Animal Care and Use Committees prior to conduct of the studies. Oral studies were conducted at MPI Research in Mattawan, MI. The protocols for these studies at MPI were reviewed and approved by MPI Research IACUC before each study. The IACUC approval ID numbers were as follows: (1) 1151-021 (mice); (2) 1151-023 (rabbits); and (3) 1151-065 (NHP). Those studies were conducted in compliance with the Testing Facility Animal Welfare Assurance (A3181-01) filed with NIH. The study in NHP did not require any procedures that were anticipated to cause more than slight or momentary pain or distress to animals, such as the collection of blood samples. NHP were observed cageside at least twice daily for any signs of morbidity, mortality, injury, and availability of food and water. Any animals found in poor health were to be monitored further for possible treatment and/or euthanasia. The IV studies in mice and rabbits were conducted at Oregon State University and the protocol approved by their IACUC for those studies was Number 3871. The IV infusion studies in NHP were conducted at Charles River Laboratories under approved protocol numbers (1) MDA00051; (2) 20002163; and (3) 20002757.

The protocols for IV infusions in NHP conducted at Charles River were reviewed and approved by PCS-NV IACUC before the study. Those studies were conducted in compliance with the Testing Facility Animal Welfare Assurance (A4112-01) filed with NIH. In an effort to minimize discomfort during the infusions, the NHP had surgery to install vascular access ports (VAP) and were acclimated to jackets that held the test article. In this way the IV infusion was carried out without the need to restrain the NHP during the process, except for brief intervals during which blood samples were taken. NHP were observed cageside at least twice daily for any signs of morbidity, mortality, injury, and availability of food and water. Any animals found in poor health were to be monitored further for possible treatment and/or euthanasia. All studies with nonhuman primates complied and followed applicable sections of the Final Rules of the Animal Welfare Act regulations (Code of Federal Regulations, Title 9), the *Public Health Service Policy on Humane Care and Use of Laboratory Animals* from the Office of Laboratory Animal Welfare, and the *Guide for the Care and Use of Laboratory Animals* from the National Research Council. The NHP studies were not terminal studies so all animals were released to their respective colonies at the end of the studies.

#### Oral Studies

ST-246 was administered by oral gavage as a methylcellulose suspension formulation with 1% Tween 80 to BALB/c mice (Charles River), New Zealand White (NZW) rabbits (Harlan), and cynomolgus monkeys (NHP, Harlan). NHP were administered ST-246 immediately after feeding to increase the bioavailability [33]. Female BALB/c mice were administered the suspension formulation via oral gavage at doses of 30, 100, 300, and 1000 mg/kg. Concentrations of ST-246 were measured by taking blood samples from three mice at each of the following time points: 0.5, 1, 2, 3, 4, 5, 6, 8, 10, 12, and 24 hours post dose. Three male and three female NZW rabbits were administered ST-246 orally as a suspension formulation at a dose of 100 mg/kg. Blood was collected at the following time points for determination of ST-246 concentration: 0.5, 1, 2, 3, 4, 5, 6, 8, 12, and 24 hours after administration. Three male and three female NHP per dose group were administered the following oral doses of ST-246 in the fed state: 0.3, 3, 10, 20, and 30 mg/kg. These doses were administered daily for 14 days. Only data from Day 1 of this study are presented here. Blood samples were collected predose and at 0.5, 1, 2, 3, 4, 6, 8, 12, and 24 hours after dose administration to measure ST-246 concentration. Blood samples were collected into Na Heparin tubes and kept on ice until the tubes were centrifuged at low speed to collect the plasma. Plasma was transferred into new tubes and stored at −70°C until bioanalysis.

#### IV infusion studies

The pharmacokinetics and tolerability of a solution formulation of ST-246 administered by IV infusion were evaluated in three animal species: female BALB/c (Charles River) and CD-1 mice (Charles River), NZW rabbits (Harlan), and cynomolgus monkeys (Charles River).

A slow push (5 minute) IV injection of a solution formulation of ST-246 was administered to a small number of catheterized female BALB/c mice at doses of 3, 30, and 100 mg/kg. Although attempts were made to collect blood samples, patency difficulties in the catheters limited the number of mice per time point. After a study confirmed that the pharmacokinetics for BALB/c and CD-1 mice were very similar (data not shown), additional IV studies were conducted in the CD-1 mouse strain. A 10-minute IV infusion of ST-246 was given via a surgically implanted jugular cannula at doses of 3, 10, 30, and 75 mg/kg to catheterized naïve female CD-1 mice. Blood samples were collected at 5, 10 (end of infusion), 20, 30 minutes, and 1, 2, 4, 8, 24 hours post dose. Blood samples for each time point were collected from three animals as terminal bleeds.

In rabbits, ST-246 was infused via the marginal ear vein at doses of 3, 30, and 60 mg/kg over a 5-minute period and at 3 mg/kg over a 15-minute period followed by blood sampling at multiple times in order to generate complete plasma concentration time curves. Blood samples were collected via the central ear artery or marginal ear vein opposite to the site of injection. Two male and two female rabbits were used for each dose group. For the 5-minute slow push IV injection, blood samples were collected at 10 minutes (5 minutes after the end of the injection), 20 and 30 min, 1, 2, 4, 8, and 24 hours after administration. Blood samples for the 15-minute IV infusion were taken at the end of the infusion (15 minutes), 25 and 45 minutes, and 1, 2, 4, 8, and 24 hours after the beginning of the infusion.

NHP were prepared for ST-246 administration by surgical implantation of a catheter in the femoral vein that was routed to a subcutaneous port. Doses of 1, 3, 10, 20, and 30 mg/kg were infused over 4 hours to groups consisting of two male and two female NHP. Two additional groups were administered the 20 and 30 mg/kg doses over 6 hours. For the 4 hour IV infusion group, blood was collected for ST-246 analysis at the following time points: 0.5, 1, 2, 4 (end of infusion), 4.25, 4.5, 5, 6, 8, 12, 16, 20, 24, and 48 hours after the start of the infusion. For the 6-hour IV infusion, the samples were collected at the following time points: 1, 2, 4, 6 (end of infusion), 6.25, 6.5, 8, 10, 12, 16, 20, 24, and 48 hours after initiation of dose administration. Blood samples were collected at multiple time points to allow complete characterization of the plasma concentration time curve and estimate the pharmacokinetic parameters. Two groups of 4 males and 4 females were used in a second study that was conducted after a 10 day washout. In the second study, the pharmacokinetic parameters were characterized over the course of a single day twice daily (BID) regimen for doses of 10 and 15 mg/kg that were infused over two 4 hour infusion periods initiated 12 hours apart. The total daily doses were 20 and 30 mg/kg, equivalent to the two highest doses that had been evaluated during both 4 and 6 hour IV infusions. For the BID study, blood was collected at the following time points for ST-246 concentration determination: 0.5, 2, 4 (end of first infusion), 4.5, 6, 8, 12, 12.5, 14, 16 (end of second infusion), 16.5, 18, 20, 24, 32, 36 and 60 hours after the beginning of infusion of the first dose. In all cases blood was collected by venipuncture using a site different than that used for dosing (not via the catheter). Blood samples were collected into Na Heparin tubes and kept on ice until the tubes were centrifuged at low speed to collect the plasma. Plasma was transferred into new tubes and stored at −70°C until bioanalysis.

### Tolerability and toxicological evaluation

Cageside observations were made throughout all of these studies for general appearance, behavior, mortality and moribundity. Preclinical evaluations for adverse events (AEs) such as vital sign measurements, physical examinations, and neurologic exams were assessed throughout the studies in NHP.

### Bioanalytical methods

ST-246 concentrations in mice, rabbit and NHP plasma were measured using a liquid chromatography-tandem mass spectrometry (LC-MS/MS) method. Blank plasma for calibration curves and quality control samples were purchased from Bioreclamation, Inc. (Westbury, NY). Two different extraction methods were used over the course of these studies. The second method, liquid-liquid extraction, was validated after the initial protein precipitation method, in order to extend HPLC column life. Both methods were validated following the FDA bioanalytical validation guidelines [38] and met FDA requirements for intra- and inter-assay precision of within 15% relative standard deviation for all validations. In one method, the extraction of ST-246 from plasma was carried out by simple protein precipitation by the addition of 9 parts methanol (450 µL) containing the isotopic internal standard to 1 part (50 µL) plasma sample. After high speed centrifugation 100 µL of supernatant was added to 200 µL of a compensation solution (0.05% acetic acid in 0.05% ammonium hydroxide∶methanol; 36∶55, v/v) and directly injected onto the LC-MS.

The second extraction method was a liquid-liquid extraction (LLE) method. Plasma samples were diluted 1∶1 with methanol containing internal standard and three volumes of water added. These mixtures were vortexed and the entire volume transferred to the extraction plate (Biotage SLE, 200 mg). Minimal vacuum was applied to load the samples and then allowed to stand for 5 minutes. Methyl tertiary-butyl ether was added to all wells (500 µL/well) and eluted with minimal vacuum. The solvent was evaporated to dryness under nitrogen (set at 50°C and 30–40 L/min). The samples were reconstituted (0.05% acetic acid and 0.05% ammonium hydroxide in methanol/water; 65∶35, v/v) by gently vortexing the plate afterwards.

The chromatographic separation was performed using a Phenyl-Hexyl column (50×2.0 mm, 5 µm, Phenomenex) with a Securityguard column, using 0.05% ammonium hydroxide and 0.05% acetic acid in MeOH/H_2_O (65∶35,v/v) at a flow rate of 400 µL/min for the mobile phase. A 3200 (or 4000) Qtrap (AB Sciex) mass spectrometer was tuned to the multiple reaction monitoring (MRM) mode to monitor the m/z transitions, 375.0/283.2 for ST-246 and m/z 341.1/248.8 for the internal standard, in negative ion mode. The MS/MS response was (1/x^2^) weighted linearly over the concentration range from 5.00 to 2000 ng/mL. The accuracy and precision of the method were within the acceptable limits of ±20% at the lower limit (5.0 ng/mL) of quantitation and ±15% at other concentrations.

### Pharmacokinetic Analysis

Pharmacokinetic parameters were analyzed with WinNonlin Phoenix version 6.1 (Pharsight, Mountain View, CA) software using noncompartmental analysis. The following parameters were estimated: terminal elimination half-life (*t*_1/2_ = ln(2)/λ*_z_*, where λ*_z_* is the first order rate constant associated with the terminal (log-linear) portion of the curve), the area under the curve (AUC_last_ = Area under the curve from the time of dosing to the last measurable concentration), the area under the curve extrapolated to infinity (AUC_0-inf_ = AUC_last_+C_last_/λ*_z_*), clearance (CL = Dose/AUC), and the steady state volume of distribution (V_ss_ = Amount in body/Concentration at steady state). The peak plasma concentrations (C_max_) and the time to peak plasma concentration (T_max_) were determined directly from the observed data.

### Statistical Analysis

Untransformed and dose-normalized data for C_max_ and AUC _0-inf_, and dose-linearity for clearance were analyzed using the JMP9.0 program (SAS Corporation, Cary, NC), which is based on the one-way analysis of variance (ANOVA) regression model, in order to evaluate dose linearity and dose proportionality. Gender differences within the same dose group were evaluated using Student's t-test. A value of *p*<0.05 was considered statistically significant.

## Results

### Mouse Studies

#### Tolerability

Preliminary bolus IV injections of ST-246 in BALB/c mice resulted in some dose-related toxicity and mortality at the highest dose of 34 mg/kg. A slower (5-minute push) IV injection resulted in some clinical signs of labored breathing and lethargy at the 100 mg/kg dose, but was well-tolerated at both 3 and 30 mg/kg. These observations suggested that the toxicity was related to the peak plasma concentration and that slower infusions would allow safe administration of higher doses. Catheterized female CD-1 mice were administered 10-minute IV infusions at doses of 3, 10, 30 and 75 mg/kg. Although mice that received the highest dose, 75 mg/kg, had an unsteady gait after the end of infusion, they recovered within 2–3 hours. All other doses were well-tolerated when administered as 10-minute IV infusions. The clinical signs occurred at the end of the infusions, at the same time as the C_max_ concentrations.

#### Toxicokinetics

**Figure 1:**
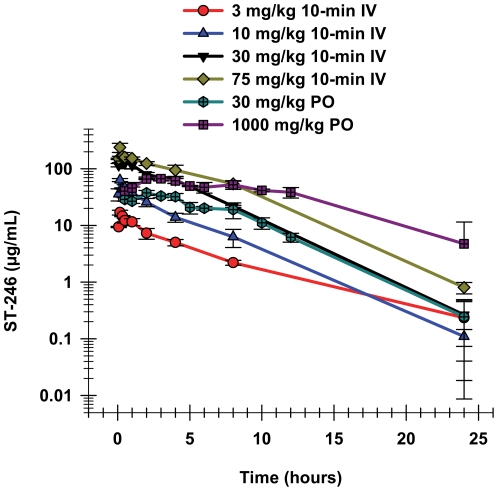
Plasma concentration time curves for oral and IV administration of ST-246 in mice. The means and standard deviations of the plasma concentrations over time are shown after oral administration of ST-246 to female BALB/c mice at doses of 30 (chartreuse hexagons) and 1000 mg/kg (purple squares). The means and standard deviations of the plasma concentrations over time after 10 minute IV infusions to female CD-1 mice at 3 (red circles), 10 (blue triangles), 30 (black triangles), and 75 mg/kg (green diamonds). Each time point is the mean value from three individual mice.

The results (Table 1 and Figure 1) show that IV infusion over 10 minutes resulted in very high C_max_ plasma concentrations of ST-246. The mean C_max_ concentration after the 10-minute IV infusion of 75 mg/kg in female CD-1 mice was 238 µg/mL, 3.6-fold higher than the C_max_ observed following a single oral administration of 1000 mg/kg, a 13-fold higher dose, in female BALB/c mice. For IV infusions, the C_max_ occurred, as would be expected, at the end of the infusions. The T_max_ for the oral doses; however, were observed later, at 2 hours post administration at all dose levels, indicating prolonged absorption in mice. Although the maximum plasma concentrations after these short IV infusions were much higher than after administration of much higher oral doses (Table 1), the exposure (AUC_0–24 hr_) was only 1.5-fold higher for the same two dose groups. Comparison of the exposure for the 30 mg/kg oral dose to the 10-minute IV infusion of the same dose showed that ST-246 had approximately 41% bioavailability for that dose. Dose-normalized exposure after oral administration declined with increasing dose, but the same trend was not observed after IV administration.

**Table 1:** Comparison of pharmacokinetic parameters for ST-246 after oral administration to female BALB/c mice and 10-minute IV infusion in female CD-1 mice (parameters at each dose were calculated from concentrations from three individual mice at each time point, thus no statistical information is available).

Route	Dose	T_1/2_	C_max_	AUC_0–24 hr_	CL
	mg/kg	hr	µg/mL	hr*µg/mL	mL/hr/kg
10-min IV Infusion	3	4.5	17	68	43
	10	2.8	64	408	25
	30	2.5	147	709	42
	75	2.8	238	1253	60

Route	Dose	T_1/2_	C_max_	AUC_0–24 hr_	CL/F
	mg/kg	hr	µg/mL	hr*µg/mL	mL/hr/kg
PO	30	2.4	38	292	102
	100	2.2	44	456	219
	300	4.1	64	669	438
	1000	4.5	66	852	1133

IV, intravenous.

PO, per oral.

The elimination half-lives were similar for the IV infusion and oral doses, those for the IV infusions doses ranged from 2.5 to 4.5 hours, while those for the oral doses ranged from 2.2 to 4.5 hours. These values were very close to what has been consistently observed throughout the oral nonclinical safety toxicokinetics studies in BALB/c mice (unpublished observation). Clearance was relatively consistent after IV infusion over the 3–75 mg/kg dose range, while the apparent clearance after oral dosing increased approximately 10-fold over the approximately 30-fold dose range. Figure 1 illustrates that even short IV-infusions in mice provided high plasma concentrations over time similar to those observed after oral administration, albeit with higher maximum plasma concentrations.

### Rabbit Studies

#### Tolerability

The tolerability and pharmacokinetics of IV administration of ST-246 was compared to that of oral administration in NZW rabbits. Although a preliminary study had shown that IV bolus administration of 1 mg/kg was well tolerated, the IV infusion studies results in mice indicated a potential for a lack of tolerability after rapid IV administration of the highest doses. Therefore, ST-246 was administered as 5-minute slow push IV injections at doses of 3, 30, and 60 mg/kg in NZW rabbits via the marginal ear vein. Whereas the 3 and 30 mg/kg doses were well-tolerated, rabbits administered the 60 mg/kg dose exhibited lethargy, labored breathing and narcosis immediately following injection. These animals appeared to recover fully 30–60 minutes after the injections. A slower (15 minute) infusion of the 3 mg/kg dose was also well-tolerated.

#### Toxicokinetics

The 60 mg/kg IV dose group had the highest C_max_ concentration, as well as the highest exposure, as measured by the AUC values (Table 2). Systematic comparison of the oral and IV C_max_ and AUC values for rabbits does not completely elucidate which parameter, C_max_ or AUC, may have been related to those clinical signs, except that the signs disappeared coincidently with the rapid decline in plasma concentrations. The 15-minute IV infusion of 3 mg/kg resulted in a mean C_max_ concentration of 5.79 µg/mL, two-fold higher than the 2.86 µg/mL observed after oral administration of 100 mg/kg (Table 2), and there were no observations in either group. Clinical signs in the rabbits were observed only at the 60 mg/kg dose, where the mean C_max_ plasma ST-246 concentration was 94.1 µg/mL, while the mean maximum plasma concentration observed for the well-tolerated 30 mg/kg dose of ST-246 was lower, at 38.5 µg/mL. Whereas the C_max_ values for short IV infusions were much higher than that of a much higher oral dose, 100 mg/kg, the exposures, as determined by the AUC measurements, were much lower. The AUC_0–24_ values observed after the 30 mg/kg dose via intravenous slow push in both genders were comparable to that recorded for the 100 mg/kg oral dosing and in spite of the high C_max_; it was evident from the cageside observations that this dose and delivery rate was well tolerated in rabbits. As was observed with mice, short intravenous infusions in rabbits produced very high maximal ST-246 concentrations, which corresponded with the time of the observed clinical signs in the animals. The pharmacokinetic parameters in rabbits were calculated using the 15-minute IV infusion of 3 mg/kg ST-246. In the 15-minute IV infusion study, blood samples were taken immediately at the end of infusion instead of 5 minutes after the end of infusion as in the initial IV infusion study. The C_max_ from the second study was therefore a more accurate reflection of C_max_ than that of the initial 5-minute IV infusion study and, in fact, the C_max_ values were substantially higher (See Table 2). The results from the single longer infusion confirmed what was observed in the shorter infusion study that evaluated a dose range; that the C_max_ values after short IV infusions were much higher than the values observed after equivalent oral doses.

**Table 2:** Comparison of pharmacokinetic parameters for ST-246 after oral administration and IV administration to New Zealand White rabbits.

Route	Dose	N	T_1/2_	C_max_	AUC_0–24 hr_	CL
	mg/kg		hr	µg/mL	hr*µg/mL	mL/hr/kg
IV Bolus	1	6	0.9±0.2	1.67±2.27	1.43±0.40	1660±2166
15-min IV Infusion	3	4	1.2±1.1	5.79±3.67	3.39±1.07	966±363
^1^IV Slow Push (5-min)	3	4	3.2±0.0	3.03±0.37	2.38±0.93	1339±521
^1^IV Slow Push (5-min)	30	4	12.2±5.8	38.5±3.7	13.3±0.7	2229±134
^1^IV Slow Push (5-min)	60	4	5.2±0.8	94.1±11.1	61.8±8.7	987±138
^1^Blood draw taken 5 minutes after actual EOI						

Route	Dose	N	T_1/2_	C_max_	AUC_0–24 hr_	CL/F
	mg/kg		hr	µg/mL	hr*µg/mL	mL/hr/kg
PO	100	6	3.7±3.2	2.86±2.03	19.8±16.6	7207±6251

EOI, end of infusion.

IV, intravenous.

PO, per oral.

**Figure 2:**
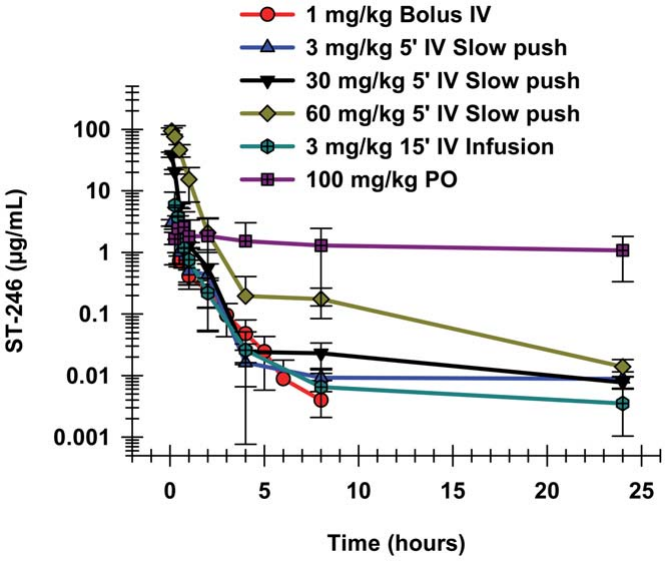
Plasma concentrations over time after IV and oral administration in New Zealand White Rabbits. Plasma concentrations of ST-246 over time are shown after oral administration of 100 mg/kg (purple squares); bolus IV administration of 1 mg/kg (red circles); or a 5- minute IV slow push of 3 (blue triangles), 30 (black triangles), or 60 mg/kg (green diamonds). A 15-minute IV infusion of 3 mg/kg (blue hexagons) is also shown. Each curve is the mean with standard deviations from two male and two female rabbits.

The semi-logarithmic graph of the plasma concentration time curves in rabbit IV infusion studies shows biphasic distribution and elimination (Figure 2). There appeared to be an initial rapid distribution phase that was followed by a slower terminal elimination phase. There was no clear dose-related trend in the elimination half-lives after IV infusion in rabbits. The elimination half-lives ranged from approximately 1 hour to 12.2 hours for the IV infusion dose groups, while the elimination half-life for the 100 mg/kg oral dose was 3.7 hours (Table 2).

### NHP Studies

#### Toxicokinetics

**Figure 3:**
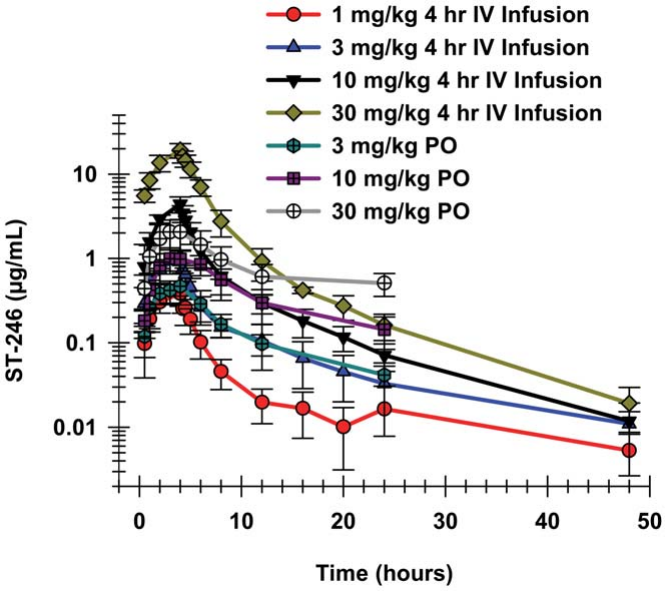
ST-246 plasma concentrations over time after oral or 4 hours IV infusions in cynomolgus monkeys. Plasma concentration of ST-246 after a single oral dose of 3 (chartreuse hexagons), 10 (purple squares), or 30 mg/kg (white circles) compared to the plasma concentration time curves after 4 hour IV infusion of 1 (red circles), 3 (blue triangles), 10 (black triangles), or 30 mg/kg (green diamonds) in cynomolgus monkeys. Each curve shows the means and standard deviations. For oral administration there were 3 males and 3 females in each dose group while for the IV infusion there were 2 males and 2 females in each dose group.

ST-246 was administered via IV infusion over 4 hours via surgically implanted vascular access ports in NHP at doses of 1, 3, 10, 20, and 30 mg/kg. The plasma concentrations increased throughout the 4-hour IV infusion of ST-246, reaching maximum concentrations at the end of the infusion (Table 3, Figure 3). The maximum plasma concentrations (C_max_) were higher after the IV infusions than after oral administration of equivalent doses (Table 3). At higher doses, the differences between the oral and IV C_max_ concentrations increased. The C_max_ concentrations after oral administration increased less than dose-proportionally, while the peak plasma concentrations after IV infusion increased more than would be expected based on dose-proportionality.

**Table 3:** Comparison of pharmacokinetic parameters for ST-246 after oral administration and IV infusions in cynomolgus monkeys.

Dose	N	T_1/2_	T_max_	C_max_	AUC_INF_obs_	CL
(mg/kg)		(hr)	(hr)	(mg/mL)	(hr_*_mg/mL)	(mL/hr/kg)
**4 Hr-IV Infusion**						
1	4	6.6±6.0	4	0.38±0.11	1.94±0.46	537±117
3	4	8.5±3.2	4	1.05±0.19	5.83±0.87	523±77
10	4	8.6±1.3	4	4.40±0.94	21.3±3.4	478±71
20	4	8.7±2.5	4	11.8±2.0	59.6±10.1	353±59
30	4	7.8±0.7	4	20.1±4.2	100±18	307±59
**6 Hr-IV Infusion**						
20	4	6.6±2.3	6	7.48±0.40	47.9±6.1	433±53
30	4	6.9±0.8	6	13.9±1.3	87.2±14.6	362±61
**BID Study 4 Hr IV Infusions SOI 12 Hours Apart - First Dose**						
10	8	N/A	4	4.59±1.29	21.0±5.0	N/A
15	8	N/A	4	7.36±1.47	32.5±5.7	N/A
**BID Study 4 Hr IV Infusions SOI 12 Hours Apart - Second Dose**						
10	8	8.9±2.5	4	5.18±0.89	26.8±5.0	429±74
15	8	9.1±2.6	4	9.08±0.95	48.7±7.5	351±44

Dose	N	T_1/2_	T_max_	C_max_	AUC_INF_obs_	CL/F
(mg/kg)		(hr)	(hr)	(mg/mL)	(hr_*_mg/mL)	(mL/hr/kg)
**PO Administration**						
0.3	6	4.0±1.3	2.5	0.054±0.028	0.38±0.15	920±437
3	6	9.9±6.0	3.3	0.496±0.145	4.53±0.49	669±65
10	6	7.0±0.9	3.8	1.08±0.28	11.8±3.7	918±275
20	6	7.2±2.3	3.2	1.71±0.71	18.3±7.0	1208±385
30	6	17.7±13.8	3.6	1.99±0.87	31.9±7.4	992±281

SOI, start of infusion.

IV, intravenous.

PO, per oral.

BID, twice a day.

The maximum plasma concentration after oral administration of ST-246 increased only 37-fold as the dose was increased 100-fold, from 0.3 to 30 mg/kg, while the exposure (AUC_inf-obs_) increased closer to the proportional increase in dose, or 84-fold. The elimination was also biphasic after oral administration, with plasma concentration time curves similar to those observed for rabbits.

The plasma elimination after IV infusion appeared to have at least two distinct phases, with a rapid distribution phase observed at the end of the infusion followed by a much slower terminal elimination phase (Figure 3). The plasma concentrations fell below the lower limit of quantitation (LLOQ = 5.0 ng/mL) before 24 hours for most of the animals in the 1 mg/kg dose group, but ST-246 was above the LLOQ for all other animals in the higher dose groups through the last time point at 48 hours.

The pharmacokinetic (PK) parameters were calculated using non-compartmental analysis for individual animals. For the IV infusions, each dose group consisted of two males and two females, while for the oral dose administration; each dose group had three males and three females. Student's t-test was performed in order to evaluate potential gender differences on the PK parameters of C_max_ and AUC_inf_. There were no statistically significant gender differences (p>0.05) with respect to the C_max_ or AUC_inf_ values at each dose level tested with a 95% confidence interval. Therefore, the mean and standard deviation values were calculated by including all animals from both genders of each dose group. The variability of individual C_max_ or AUC_inf_ values within each dose group was quite small, with the exception of one or two animals that had inadvertent and obvious subcutaneous injections and whose values were excluded from group means (individual data not shown).

Although the C_max_ and AUC_inf_ values increased dose-proportionally as the 4 hour IV-infused doses increased from 1 to 10 mg/kg, the increases in these values were greater than dose-proportional at the 20 and 30 mg/kg doses (Table 3). The C_max_ values for the 3 and 10 mg/kg doses were 2.7-fold and 11.5-fold higher, respectively, than that of the 1 mg/kg dose, while the corresponding values for the 20 and 30 mg/kg doses were 31-fold and 52-fold higher, respectively. The AUC_inf_ values increased 3.0, 11.0, 32, and 53-fold higher for the 3, 10, 20, and 30 mg/kg doses, respectively, compared to the 1 mg/kg dose. The increase in exposure above dose-proportionality was also reflected in the strong trend of decreased clearance as the dose infused over 4 hours was increased (Table 3). Extending the IV infusion length to 6 hours for the 20 and 30 mg/kg doses increased the clearance (and decreased exposure) relative to the shorter infusions. The clearance values for the longer infusions of the higher doses, however, were still lower than the 1, 3, and 10 mg/kg doses. The apparent decrease in clearance values with increasing doses were not statistically significantly different (p>0.05) when evaluated by ANOVA.

The mean C_max_ plasma concentrations were higher for the 4 hour infusions compared to the 6 hour infusions by approximately 50%, and the exposures calculated for shorter infusions were also higher, although only by approximately 20%. Plasma concentrations after the end of infusions appeared to have at least two phases for all IV infusions, with a rapid distribution phase clearly observed just after the EOI followed by a slower terminal elimination phase. The plasma concentration time curves appeared similar for the two infusion rates and doses, except for the T_max_ and actual plasma concentrations.

The elimination half-lives after IV infusions were relatively constant over the dose range and different lengths of infusions, ranging from 6.6 to 9.1 hours (Table 3). Oral administration of the 30 mg/kg dose resulted in a 17.7 hour terminal elimination half-life, compared to a 9.9 hour half-life for the orally administered 3 mg/kg dose. (This longer half-life was due to a single animal that had a very long value. If the value for that animal was removed the mean elimination half-life for the remaining five NHP would have been approximately 10 hours.) Oral administration of doses of up to 20 mg/kg had similar elimination half-lives; and these elimination half-lives were very similar to those observed after IV infusions (Table 3).

**Figure 4:**
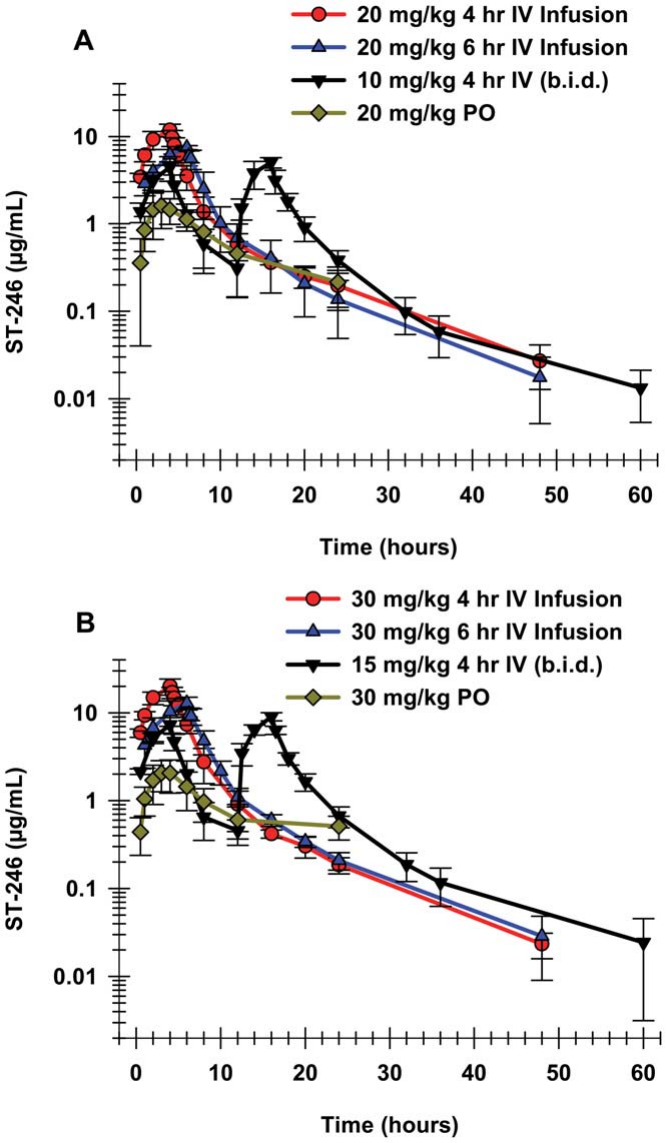
Exposure after different dosing regimens of either 20 or 30 mg/kg ST-246 to cynomolgus monkeys. The mean and standard deviation values for the plasma concentrations over time are shown for different dosing regimens of (A) 20 mg/kg or (B) 30 mg/kg. The dosing regimens included oral administration (3 males and 3 females in each dose group, green diamonds), 4 hour IV infusion (2 males and 2 females in each dose group, red circles), 6 hour IV infusion (2 males and 2 females in each dose group, blue diamonds), and BID two 4 hour IV infusions initiated 12 hours apart (4 males and 4 females in each dose group, black diamonds).

A single day twice-a-day (BID) administration study of the two highest total daily doses was conducted via two 4 hour IV infusions initiated 12 hours apart over a single 24 hour time period (Figures 4A and 4B). The individual doses were 10 and 15 mg/kg, so that the total daily doses were 20 and 30 mg/kg/day for the two dose groups, respectively. Plasma concentrations increased over each of the 4 hour IV infusion periods with the C_max_ for most animals occurring at the end of the infusion. At the last time point, 60 hours after the beginning of the first IV infusion dose, the ST-246 concentration was quite close to the lower limit of quantitation (5 ng/mL) for all of the animals in both dose groups. The semi-logarithmic graphs (Figures 4A and 4B) suggest that ST-246 elimination from the plasma after the end of the second infusion was at least biphasic, with a rapid distribution after T_max_ clearly observed, as well as a slower terminal elimination phase for both doses.

The BID administration study had 4 males and 4 females in each dose group, providing a larger number with which to evaluate any potential gender differences in the pharmacokinetic parameters after IV infusion. A student's t-test analysis of the PK parameters (C_max_, AUC_last_ or AUC_inf_, Cl and V_ss_) showed equivalence for the two genders, with the exception of the C_max_ observed during the first phase of dosing at the 10 mg/kg/dose level (p<0.05). Because there were no consistent differences between the pharmacokinetic parameters for the two genders, the final mean and standard deviation values for ST-246 were calculated by combining the data from both genders for each dose group.

The C_max_ and AUC_last_ values for the 15 mg/kg dose were 1.6-fold higher than those of the 10 mg/kg dose during the first 4 hour IV infusion. During the second IV infusion the increase was slightly more, approximately 1.8-fold for the both C_max_ and AUC values. The terminal elimination half-lives, calculated from the second infusion, were essentially identical, 8.9 and 9.1 hours for the two doses, respectively. Clearance was also essentially equivalent for these two doses and within the range observed for the single IV infusions.

#### Tolerability

As was also observed in the IV infusions studies in mice and rabbits, rapid infusions of the highest doses in NHP, 30 mg/kg infused over 4 hours, resulted in clinical signs, coincident with the end of the infusion. Three out of four animals that received the 30 mg/kg dose of ST-246 over the 4-hour infusion duration exhibited slight generalized tremors. These tremors were observed within 13 minutes of the end of the infusion on the day of dosing and resolved approximately 2 hours after the end of the infusion, indicating reversibility of this toxicity. Tremors were not observed in animals dosed at 30 mg/kg over 6 hours or in any of the animals that received the 20 mg/kg dose via either infusion duration. In addition, no clinical signs were observed throughout the BID study in any of the NHP. The mean peak plasma concentration for the 30 mg/kg 4-hour infusion group was 20.0 µg/mL, while the mean peak plasma concentration for the same dose infused over 6 hours was approximately 13.0 µg/mL. The peak plasma concentrations were much lower in both 20 mg/kg dose groups, as well as the BID study (Table 3).

## Discussion

The antiviral efficacy of ST-246 against poxviruses has been demonstrated after oral administration in mice, rabbits, ground squirrels, prairie dogs, and NHP [24], [28]–[32], [34]. The pharmacokinetics of ST-246 after oral administration has been thoroughly characterized in mice, NHP and humans, with limited information in rabbits, rats, and dogs. A complete understanding of the pharmacokinetics is important in species in which the efficacy is also being evaluated, as the selection of the human therapeutic dose will necessarily be chosen based on the animal PK/PD relationship, due to the lack of evaluable orthopox disease in humans.

The similarity of the plasma concentration time profiles after oral and IV administration demonstrated that IV administration of a dose of ST-246 should provide efficacy against orthopoxviruses, assuming the administration is slow enough to avoid what appeared to be a C_max_-related toxicity. Oral administration of 100 mg/kg provided optimal efficacy in mice against ectromelia virus [28]. Exposure after the oral 100 mg/kg doses was close to that measured after the 10 mg/kg IV slow push administration (Table 1), indicating a reasonable dose at which to start to evaluate antiviral activity with the IV formulation. Elimination in mice appeared to be mono-exponential after oral administration, but appeared to have a very short and rapid distribution phase after IV administration. Oral administration of ST-246 in mice had not elicited any dose-limiting toxicity at doses of up to 2000 mg/kg, although this might have been due to the fact that absorption after oral administration appeared to be saturated and higher doses in particular did not result in concomitantly higher peak plasma concentrations and exposure. The observed dose-limiting toxicity of unsteady gait and disequilibria after IV administration in mice, which was observed briefly at the end of the IV infusion, and that resolved within an hour, suggested that the toxicity might be related to the maximum plasma concentration. This same type of toxicity was observed in the rabbit IV infusions, in which the 5 minute infusion of 60 mg/kg was the maximum-tolerated dose. At the end of the infusion of the 60 mg/kg dose, lethargy, labored breathing and narcosis were observed. All animals appeared to fully recover within 30–60 minutes after the end of the infusion, again, coincident with the rapidly decreasing plasma ST-246 concentrations. Oral administration had not elicited any dose limiting toxicity at 100 mg/kg in rabbits. In NHP, mild ataxia was observed in three out of four animals at the end of the 4 hour IV infusion of the 30 mg/kg dose, but in none of the other doses or dosing regimens. In fact, ST-246 had been administered orally daily at 300 mg/kg for as long as three months and had been well-tolerated at that dose. As was observed in mice and rabbits, the clinical signs were observed only at the end of the infusion of the highest dose. In NHP this was at the 30 mg/kg dose administered over 4 hours, coincident with the peak plasma concentrations, and resolved after a short period of time.

Taken together, the observations of clinical signs at peak plasma concentrations in mice, rabbits, and cynomolgus monkeys after IV infusions of the highest dose level over the shortest time period and resolution of these toxicities coincident with the decrease in plasma concentrations strongly indicate that this observed toxicity was related to the high peak plasma concentrations. Further, the toxicity appears to be reversible, and was not observed when the plasma concentrations were kept at lower concentrations by slower infusion of equivalent doses of ST-246. Although the mechanism of this toxicity is not yet known, the same ataxia was previously observed after oral administration of 1000 and 2000 mg/kg doses in NHP, where the mean C_max_ was approximately 20 µg/mL, similar to that observed after the 4-hour IV infusion of 30 mg/kg ST-246. This CNS toxicity was also observed at lower doses in the dog, where the maximum-tolerated dose for repeat dose administration for ST-246 was 30 mg/kg [34]. A comparison of the ST-246 concentrations in the CSF and brain between NHP and dogs after comparable doses showed that the concentrations were much higher in the dogs, possibly explaining the unique sensitivity. In each of the species where this toxicity was observed, further investigations demonstrated that slower infusions eliminated the clinical observations, indicating that IV infusions in humans can be conducted safely by initiating any studies with low doses administered as slow IV infusions.

The plasma concentration time curves in rabbits dropped very rapidly after the end of the infusion compared to what had been observed after oral administration, where apparently prolonged absorption provided a long terminal elimination phase with relatively high concentrations after a single oral administration of 100 mg/kg (Figure 2). Interestingly, as the IV infused dose was increased from 30 to 60 mg/kg, the concentration observed during the terminal elimination phase increased, suggesting that higher doses may have, as was observed in NHP, saturated some mechanism of clearance. The rapid decrease in plasma concentrations in rabbits after the end of the infusions suggests prolonged infusions might be required for efficacy studies in rabbits. Additional infusions studies would be needed to confirm the potential relationship between administered dose and clearance in rabbits.

The oral ST-246 study in NHP evaluated the pharmacokinetics over a dose range which encompassed those used in efficacy studies, from 0.3 to 30 mg/kg. The results demonstrated that absorption appeared to be saturated as the orally administered dose was increased, and this was reflected in both the C_max_ concentrations as well as the exposure. Although the C_max_ as well as the exposure increased over this oral dose range, they increased less than dose-proportionally. The C_max_ increased only 37-fold over the 100-fold dose increase, while the exposure, as measured by the AUC_inf_, increased 84-fold, much closer to the 100-fold dose increase.

The saturation of absorption, which led to decreased plasma concentrations and exposure with increasing oral doses, would not be observed after IV infusions, where absorption is not a component of the pharmacokinetics. The bioavailability of ST-246 in NHP based on comparison of identical oral and IV doses thus ranged from 77% at 3 mg/kg to 31% at 20 and 30 mg/kg doses. After IV infusions, the exposure at these high doses was actually higher than would be expected based on dose-proportional exposure. The exposure for the 4 hour IV infusions of 20 and 30 mg/kg were 30-fold and 50-fold higher, respectively, than the exposure observed after the 1 mg/kg IV infused dose (Table 3). Longer infusions reduced the C_max_ values closer to dose-proportional for the 20 and 30 mg/kg doses, while the AUC values decreased to 25-fold and 45-fold higher than the exposure observed for the 4 hour 1 mg/kg IV infusion (Table 3). The BID dose regimen confirmed the observation that slower infusions decreased not only the C_max_, but reduced the total exposure values to closer to dose proportional. These results suggest that a rapid rate of infusion of ST-246 may have saturated some clearance mechanism. Over a similar dose range, oral absorption may have decreased with increasing dose, so that clearance remained relatively constant, or even increased slightly.

The plasma concentration time curves in NHP after oral administration were very similar to those observed after both the 4 and the 6 hour IV infusions, except for the higher peak plasma concentrations observed after IV administration. The similarity in the elimination half-lives, as well as the similar plasma concentrations during the terminal elimination phases, suggest that similar efficacy could be achieved.

Visual inspection of plasma concentration time curves after oral administration of ST-246 suggests that absorption was prolonged and may have some impact on the apparent elimination half-lives. However, the elimination half-lives did not change significantly for any of the three species studies between oral and IV administration, indicating that prolonged absorption did not play a significant role in the elimination half-lives after oral administration. Given these similar elimination half-lives across all three species examined by oral and IV infusions, it appears that longer IV infusions should be administered in order to reduce the high plasma concentrations, and to avoid the coinciding toxicity, while continuing the once daily dosing regimen that is currently being used in oral studies.
